# Development of Teeth

**Published:** 1891-06

**Authors:** Will. H. Whitslar


### Development of Teeth.

BY WILL. H. WHITSLAR, M.D., D.D.S.

Read before the Northern Ohio Dental Association, at Oberlin, O., May 12th, 1891.

The first general conception with which we should begin the study of vital phenomena is the mutual dependence of all things, which in their groupings together, compose the system of nature. This dependence requires for each being the simultaneous existence of all the others. Physiology is an independent science resting upon truths of its own, which it draws from the observation of those actions, which in their aggregate connections constitute life, but it is enriched by facts furnished to it by collateral sciences. Of all collateral studies anatomy is that which will enable us to obtain the best appreciation of the beauties of living phenomena. Anatomy is, indeed, a part and parcel of physiology, bearing the same relation to it as geography does to history. Now, the phenomena of development, growth, sensation, decay and death, and many others, belong to life, but life occurring only in material structures which subsist in obedience to the laws of physics and chemistry, is truly a superstructure on these laws, and can not be studied independently of them. Indeed, the greater part of the phenomena of organic beings are chemical and physical phenomena, modified only by an additional principle—*life*. When asked what is life, all we can do is to tell some characteristics of the principle of life. One says it is the union of soul and body; another that it is the inherent principle of action, but I think it may be tersely summed up in this: "Life is the visible expression of that arrangement in nature by which elementary substances are in such wise combined as to form organic bodies."

The beginning of all things is small, and the neglect of small things is the grand error in studying life as well as in starting life, and I might add this aphorism: "The nearer to the beginning that the deviation from the right course is, the greater the wrong in the end."

To begin aright, then, we must recognize a fact that was unknown for many centuries to anatomists and physiologists, and that is this; *All animals arise from eggs.*

The human body, like every other animal organism, is composed of a vast number of units called cells, all of which originate from a single form called the parent cell; to give its scientific name it is termed *cytula.* I do not mean that all cells are originally from a single form in outline, but in substance we reproduce only our kind; there is no evolution from one organized being to that of another different in form and duty. Some authorities say that it is more proper to call a cell a corpuscle or plastid.

The parent cell, or cytula, is the result of the union of the spermule, or paternal cell, with the ovule, or maternal cell. The union of these two elements is commonly spoken of as the impregnation or fecundation of the ovum.

(Illustrated on blackboard.)

Following impregnation the process of segmentation takes place by which the egg divides and sub-divides by a series of successive segmentations into a number of cells of which all the cells of the future animal are the direct descendents. We must, however, keep in mind that whilst the whole body of matter is called an egg, yet only a part is the true egg, and the remaining part forms, or is used, as food by the growing germ. It is only the *germ spot* that divides, and this spot is generally known in scientific parlance as the *germinal disc.* In birds the egg is large, and the larger part is used for food on account of its being unable to segment. In mammals the ovum is small and contains a small amount of food yolk and in consequence the whole ovum segments. Partial segmentation is known as *meroblastic*, and when the whole egg segments it is known as the *holoblastic type.*

Now, it would be impossible for me to give in one dissertation on development of the teeth even a synopsis of segmentation and its sequelæ resulting in the perfected animal, so let it suffice to say, that consequent upon segmentation there are formed three layers of cells known as the hypoblast, epiblast and mesoblast.

From these layers of cells, intricate and mysterious but yet beautiful developments occur, giving rise to parts whose aggregate co-ordinations reveal the living man. We now learn that the enamel of a tooth is a derivative of the epiblastic layer, whilst all the remaining parts are of mesoblastic origin. The enamel then comes from that layer which forms the epidermis, retina of the eye, labyrinth of the ear, taste buds, crystalline lens and the lining membrane of the mouth. The dentine, cementum and pulp of the tooth takes origin from the same layer that composes the bones, muscles, arteries, veins, lymphatics, generative and urinary organs, connective tissue, etc.

It would be proper to describe the process of the development of the jaws and buccal cavity, and it would conduce to our understanding of the development of teeth, as well as how enamel and dentine are deposited, but time and space forbid. It involves a minute study of histology as well as of crystallography.

Now, dental histologists tell us that at about the forty-fifth day of embryonic life, in the epiblastic layer which covers the gums, is seen the first indication of cellular activity which will result in the evolution of the teeth. The thickening of the epithelium is at first general, but after a short time it becomes thicker at the crest of the gums and in a line of the future arch of the teeth.

As the outer cells are an elaboration, or a kind of casting off from the younger cells which have performed their active work of life, we find that, as a result of the activity of the younger cells, or infant layer of the reta malpigii, the older cells proliferate so as to form a ridge. At the same time these cells are heaped up, the infant layer is pushed down, pushing the subepithelial layer beneath.

This gives rise to the opinion of Goodsir that there was first formed a groove, called primative groove. Naturally this would seem so if the older cells were taken away leaving a groove, as it were, in which they rested. All these cells together as they repose in the so-called groove have been given several names, as *bourrelet*, which means pad or cushion, or *cartilago dentalis*, because of it having been supposed to be cartilage. This is now called the *epithelial band*, because it expresses the condition of affairs better.

This band assumes a direction with its axis toward the inner side of Meckel's cartilage, and the next thing to notice is the peculiar curve of the band whose convex side is always toward the outer side of the jaw. The whole band is, however, on the inner side. At the anterior part of the jaw it is deepest and narrower, and gradually grows shallower until it flattens out into the epithelial covering of the jaw.

In the deeper parts of the band, cellular activity is greater, and here cells proliferate to form a process of the band, and spreading out into a sheet-like form, it is termed the *lamina*. As the lamina grows deeper the band becomes shallower, and sometimes disappears. From the lamina at regular periods corresponding with the position of the future tooth, small buds appear and extend into cords, each cord developing, in time, into the enamel organ of the temporary tooth. As said before, the cord is a process of the lamina and like it, is composed of a solid ingrowth of cells which constitute the lamina. The infant layer constitutes the outer layer of the cord and is composed of oval and spheroidal cells. As the cord grows, cells inside of it proliferate to form a bulbous extremity. Then the part of the cord connecting the bulb with the lamina is known as the neck of cord. The length of the cord varies. In the human being it is short. For the permanent teeth the cords are longer than for the temporary teeth. It is well to keep in mind that the cord is derived from the lamina, and the lamina is from the band, and the band from the oral epithelium. Remember too that the *infant* layer of cells constitutes the outside layer of the cord.

Speaking of the cord it might be well to speak of the cord for the permanent teeth. The cord for the permanent tooth arises from the cord of the corresponding temporary tooth, that is, from the central and lateral incisors, bicuspids, and in some instances, from the first permanent molar. As a rule the cord for the permanent molars arises from the Malpighian layer of the mucous membrane and from the lingual aspect of the temporary teeth.

The time for the origin of the cord for the permanent teeth varies considerably. The length of the cord also varies, and has a spirility that marks it as a characteristic of the cords for the permanent teeth.

As the cord grows down the end becomes bulbous and it invaginates because of its contact with a papilla which is derived from the connective tissue which will be remembered as a derivative of the mesoblast.

The growth of the papilla is upward toward the gum, whereas the cord has been downward into the substance of the jaw. The bulb of the cord then becomes known as the *enamel organ.*

Now this enamel organ has been and still continues to be the source of much discussion. All agree that this bulbous part of the cord or enamel organ is the means by which enamel is deposited, but just how it is performed remains undecided by all authorities. At the Union Dental Meeting in Boston recently, Dr. R. R. Andrews, of Cambridge, Mass., who is now one of our best microscopists, in a paper on the Development of Enamel, states, "That the cells of the internal epithelium of the enamel organ, the ameloblasts, contain in the part nearest the calcifying tissue large numbers of minute glistening bodies, which have been misnamed granules, but which are really calcospherites." And he further states that these little globules are given out by the enamel-cell into protoplasmic substance and there a condition known as caloglobulin is formed, which in turn calcify, forming rods, and the protoplasmic substance which surrounds them becomes known as the cement substance.

Prof. Sudduth from whom I have largely drawn this description regards the enamel organ as a store-house for the calcific material of which the enamel is composed, and that the stellate reticulum, or that part which is contained in the bulb of the cord, is the essential agent in the formation of enamel, and that the ameloblasts, or that layer of cells to be found on the part of the bulb which invaginates. are the agents which, like men laying brick, deposit the granules in many columns which form the enamel. In this enamel organ there seems to be about enough calcific matter to form the first layers of enamel. Afterwards the outer cells of the enamel organ disappear and capillaries are found nearer the ameloblasts, so that accordingly the lime salts may almost directly be deposited from the blood. Here is an important item to consider, for by this knowledge we can almost infer that infectious and zymotic diseases may arrest development resulting in those markings we so often perceive.

As ameloblasts are to enamel, so are the odontoblasts to dentine; for the papilla is surrounded by a layer of cells known as odontoblasts, and these cells operate in a similar manner as do ameloblasts in depositing the calcific material. As depositions of dentine take place, the papilla grows smaller and eventually becomes what is known as the dental pulp. It is worthy of note that there is no connection whatever between the enamel organ and the dentinal papilla by blood vessels or other tissues and consequently none between their products. At the same time that the dentinal papilla begins to grow there also begins to form around it an envelope of tissue, coming as a derivative also of the mesoblast, and the exact office of this envelope, so to speak, is not known, but is supposed to be instrumental in forming the cementum. This forms at the same time that the periosteum for the bones is in progress. As time progresses all these processes unite to make the formed tooth, and this illustrates our proposition in the beginning, of the mutual dependence of all things, and this substantiates the truth that no effect is the result of any one cause, except the first Great Cause.

In conclusion, allow me to say that it must be acknowledged that this subject has only been treated in a summary way in this paper. A paper complete in all details cannot now be written, since the cycle of our knowledge remains immature. This cycle began in the year 1574, when Eustach discovered the tooth germ and 1815 when Delabarre gave the first details of the development of the teeth. Within this cycle of experimentation and discovery we have to notice the names of Legro and Magito, Tomes, Huxley, Hunter, Desirabode, Sudduth, Beale, Andrews, Black, Graf, Spee, Tomes, Boedecker, Heitzman, Abbot, and many other luminaries whose contributions have almost completed the cycle, but ere it is completed all must unite in definite conclusions.

